# Forefoot Centre of Pressure Patterns in Black Male African Recreational Runners with Pes Planus

**DOI:** 10.3390/jfmk10030273

**Published:** 2025-07-16

**Authors:** Jodie Dickson, Glen James Paton, Yaasirah Mohomed Choonara

**Affiliations:** 1 Department of Chiropractic, University of Johannesburg, Johannesburg 2006, South Africa; 2 Department of Podiatry, University of Johannesburg, Johannesburg 2006, South Africa; yaasirahc@uj.ac.za

**Keywords:** pes planus, centre of pressure, gait biomechanics, Black African runners, foot posture

## Abstract

**Background:** Pes planus is a condition where the arch of the foot collapses, resulting in the entire sole contacting the ground. The biomechanical implications of pes planus on gait have been widely studied; however, research specific to Black African populations, particularly recreational runners, is scarce. **Aim:** This study aimed to describe the forefoot centre of pressure (CoP) trajectory during the barefoot gait cycle among Black African recreational runners with pes planus. **Methods:** A prospective explorative and quantitative study design was employed. Participants included Black African male recreational runners aged 18 to 45 years diagnosed with pes planus. A Freemed™ 6050 force plate was used to collect gait data. Statistical analysis included cross-tabulations to identify patterns. **Results:** This study included 104 male participants across seven weight categories, with the majority in the 70-to-79 kg range (34.6%, n = 36). Most participants with pes planus showed a neutral foot posture (74.0%, n = 77) on the foot posture index 6 (FPI-6) scale. Flexible pes planus (94.2%, n = 98) was much more common than rigid pes planus (5.8%, n = 6). Lateral displacement of the CoP was observed in the right forefoot (90.4%, n = 94) and left forefoot (57.7%, n = 60). Load distribution patterns differed between feet, with the right foot favouring the medial heel, arch, and metatarsal heads, while the left foot favoured the lateral heel, medial heel, and lateral arch. No statistical significance was found in the cross-tabulations, but notable lateral CoP displacement in the forefoot was observed. **Conclusions:** The findings challenge the traditional view of pes planus causing overpronation and highlight the need for clinicians to reconsider standard diagnostic and management approaches. Further research is needed to explore the implications of these findings for injury prevention and management in this population.

## 1. Introduction

Running plays a significant role in the global sports landscape, with African athletes particularly from East and Southern Africa consistently dominating middle- and long-distance events. While elite-level performance has received extensive attention, there is growing global participation in recreational running across African populations. This broader engagement underscores the need for biomechanical studies that consider both high-performing and recreational athletes within African contexts.

The foot is categorized into three postures: (1) the normal arched foot, (2) the pes planus foot, and (3) the high-arched foot [1]. Pes planus is a common musculoskeletal condition characterized by the collapse of the medial longitudinal arch of the foot, leading to the entire sole contacting the ground [2]. This postural presentation can be congenital, i.e., paediatric pes planus, or acquired, i.e., adult pes planus [3]. Pes planus can be further subdivided into two functional types: flexible or rigid pes planus [4]. Flexible pes planus is a frequently encountered condition attributed to abnormalities in soft tissues, including ligamentous laxity and muscular dysfunction [5,6]. Rigid pes planus occurs less frequently but forms due to underlying congenital coalition, arthritis, severe soft tissue contractures, or structural abnormalities [7]. Pes planus, characterized by a lowered arch, disrupts normal biomechanics through compensatory movements, ultimately resulting in abnormal foot-loading patterns [8,9]. Pes planus is commonly known to increase the pronator moment with an increased load transmitted towards the medial aspect of the foot and first metatarsal [10]. These biomechanical changes can significantly affect athletic performance, especially in runners, where the repetitive loading forces exerted on the feet are considerable [11]. Additionally, these altered biomechanics have been linked to increased risk for injuries such as plantar fasciitis, tibialis posterior tendinopathy, and stress fractures [12,13]. Despite the global and African prominence in running, historical research on pes planus and gait biomechanics has disproportionately focused on Caucasian populations [14]. These studies frequently associate pes planus with overpronation, typically characterized by excessive medial displacement of the foot’s centre of pressure (CoP) during the gait cycle [15]. However, there is limited understanding of how these patterns may present in African populations, particularly in recreational runners. Some studies have described population differences in foot morphology such as lower arch heights or broader forefoot dimensions in Black African individuals. Given that Africa is underrepresented in this domain of biomechanical research, it is imperative to examine these population-specific characteristics to guide evidence-based interventions. Notably, this study focuses on gait rather than running due to the technical challenges of accurately capturing CoP data during dynamic running conditions, including higher speeds, greater ground reaction forces, and reduced foot–platform contact time. These factors can compromise the spatial and temporal resolution of pressure measurement systems (e.g., force plates or instrumented treadmills), making gait analysis a more controlled, replicable, and valid proxy for studying foot-loading patterns relevant to running biomechanics.

Some findings suggest that Black African individuals may exhibit unique foot morphologies, including lower arch heights and wider forefoot dimensions [16,17], potentially resulting in different CoP trajectories and gait compensations compared to non-African populations. Given that Africa is underrepresented in this domain of biomechanical research, it is imperative to examine these population-specific characteristics to guide evidence-based interventions. To address these gaps, it is essential to investigate gait biomechanics specifically in Black African recreational runners with pes planus. Such targeted research can clarify whether existing assumptions about overpronation and CoP shifts apply uniformly across populations, or if alternative compensatory patterns are present.

Despite facing similar repetitive loading forces, recreational runners with pes planus are often overlooked in biomechanical research compared to their elite counterparts. Yet, understanding their specific gait patterns is vital for developing appropriate clinical interventions and injury prevention strategies, particularly as recreational running continues to grow in popularity worldwide [18,19]. Although most existing studies concentrate on elite athletes, evidence suggests that recreational runners also exhibit gait abnormalities that warrant focused analysis [20]. The CoP represents the point where the ground reaction force is exerted on the plantar aspect of the foot [21]. The CoP is closely linked to the body’s centre of mass and centre of gravity, providing critical insights into gait dynamics [22]. During gait, the CoP moves in a heel-to-toe direction, representing the gait line [22]. Small displacements of the CoP towards either the medial or lateral side can be observed using pressure platform devices [23]. If the CoP displaces towards the medial aspect of the forefoot, it is typically due to an increase in the pronatory moment in the foot, whereas a lateral displacement indicates supination [10]. In individuals with pes planus, the CoP is typically expected to shift medially due to the collapsed medial longitudinal arch, resulting in overpronation [24]. However, there is limited understanding of how these patterns may present in African populations, particularly in recreational runners. However, some studies have found that the pes planus CoP was more laterally displaced in the forefoot compared to high-arched and normal-arched feet, suggesting a more complex relationship than traditionally understood [10,25,26]. Accordingly, this study explicitly tests the hypothesis that recreational runners with pes planus will demonstrate either a medial shift in CoP trajectory consistent with overpronation, or alternatively, a lateral displacement pattern reflecting different compensatory mechanisms. By formulating this hypothesis, we aim to clarify the nature of CoP shifts in this population and provide a basis for interpreting our findings within a hypothesis-driven framework. Notably, this study focuses on gait rather than running due to the challenges in capturing accurate CoP data during dynamic running. Gait analysis allows for controlled and replicable conditions that still reflect important postural and biomechanical adaptations relevant to running performance and injury risk. The objective of this study is to examine CoP trajectories in Black African recreational runners with pes planus. Specifically, it aims to determine whether the expected medial shift in CoP occurs, or whether different compensatory mechanisms are evident. Addressing this question may provide critical insights into population-specific biomechanics and help inform more appropriate orthotic and clinical strategies. These findings could inform more effective treatment strategies, as standard orthotic solutions targeting overpronation may not be suitable for individuals with lateral CoP patterns. The study emphasizes the need for ethnic and biomechanical diversity in clinical research, ensuring tailored and relevant interventions across populations [27,28].

## 2. Materials and Methods

### 2.1. Study Design

The research employed a prospective, explorative, and quantitative design to analyse the forefoot centre of pressure (CoP) trajectory in Black African recreational runners diagnosed with pes planus. A portable Freemed™ 6050 force plate (The FreeMed 6050 force plate utilizes freeStep™ software (version 2.1), which is also developed by Sensor Medica Srl in Via Bruno Pontecorvo, Italy) was utilised to record and analyse gait patterns specific to this population group [24]. Data collection occurred over a five-day period between 3 and 25 February 2024. An explorative approach was selected to investigate the distinctive biomechanical characteristics associated with pes planus in this under-researched demographic [25]. The quantitative methodology enabled the collection of objectives, numerical data necessary for measuring and statistically analysing the forefoot CoP trajectory during the stance phase of the gait cycle [24].

To ensure methodological rigour, a total of 182 individuals were initially screened for eligibility based on inclusion criteria such as age, running status, absence of lower limb injury, and clinical diagnosis of pes planus. After applying the exclusion criteria which included recent musculoskeletal injuries, prior foot surgery, or neurological disorders, 78 individuals were excluded. Reasons for exclusion included insufficient clinical indicators of pes planus, presence of lower limb pathology, or failure to meet the physical activity threshold. This resulted in a final sample of 104 eligible participants, all of whom met the study requirements and consented to participate.

### 2.2. Participants

The study recruited 104 male Black African recreational runners between the ages of 18 and 45 years. Participants were required to meet the following inclusion criteria:

- Biological males only, to eliminate biomechanical and gait variability associated with gender. Females typically demonstrate structural and functional differences in the hip and knee joints, influencing lower limb biomechanics [25].
- Black African population affinity. This demographic was selected due to the paucity of research exploring foot biomechanics and posture in this group [26]. Participants were not classified according to ethnic subgroups (e.g., Zulu, Xhosa) but instead homogenized as Black African based on population affinity. According to Statistics South Africa [22], Black Africans represent approximately 81% of the South African population yet remain underrepresented in biomechanical literature [10,17].
- Age range between 18 and 45 years. This criterion was used to minimize age-related biomechanical changes and degenerative processes, such as altered plantar pressure distribution associated with musculoskeletal and sensory decline in older individuals [29].
- Participation in at least one of the designated recreational road-running race events.
- Presence of pes planus in one or both feet, as confirmed through clinical evaluation.
- No history of lower limb surgery, recent lower limb injuries, or current foot pain.

Participants with unilateral or bilateral pes planus were included. Although compensatory forces may vary between these subtypes, both presentations are clinically relevant and represent the real-world distribution of the condition among runners. Future studies may consider stratifying the data to account for this difference. To control for confounding effects, participants’ years of running experience were recorded during data collection and considered during data interpretation. A study limitation is that leg dominance was not controlled, which may affect left–right CoP asymmetry. This should be considered when interpreting the results. All participants underwent a standardized clinical foot evaluation to confirm the diagnosis of pes planus and assess foot posture and type. The evaluation included the following step-by-step procedures:

1. Visual inspection of the medial longitudinal arch during relaxed standing and non-weight-bearing positions.
2. Navicular drop test to assess arch collapse.
3. Assessment of foot flexibility (to differentiate between flexible and rigid pes planus).
4. Application of the foot posture index 6 (FPI-6), a validated clinical tool used to assess foot alignment across six anatomical and functional criteria.
5. Photographic and observational comparison with a neutral foot model.
6. Manual palpation and joint mobility assessment to rule out rigid foot deformities.

The FPI-6 has been validated in multiple populations and is considered reliable for both clinical and research settings [16]. A customized foot examination form, developed specifically for this study, was derived from validated sources, including the FPI-6 and the navicular drop test. The form was piloted on a small group of non-participant volunteers to ensure clarity and relevance. Data from the pilot study were excluded from the final analysis.

Exclusion criteria included:

- Neurological conditions affecting gait or balance.
- Use of orthotic devices within the past six months.

A study limitation is that leg dominance was not controlled, which may affect left–right CoP asymmetry. This should be considered when interpreting the results.

### 2.3. Data Collection

**Figure 1:**
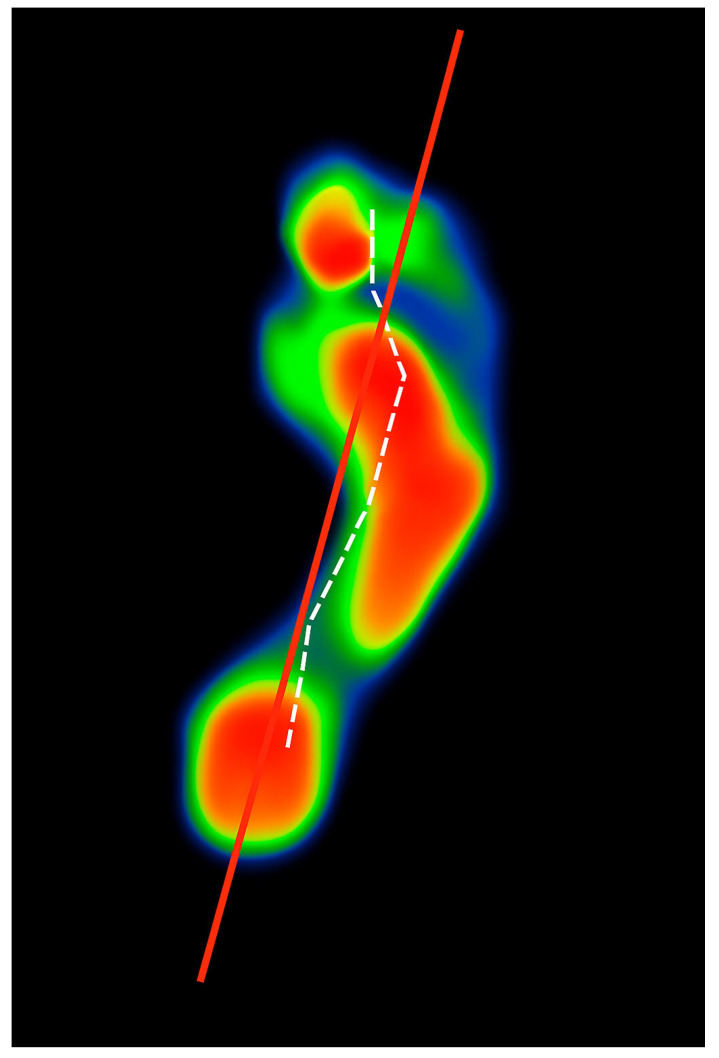
Barometric pressure of a right footprint with the CoP path indicated as the white dashed line. The red solid line divided the foot through the midline of the 2nd metatarsal. The CoP white dashed line is on the lateral aspect of the red line, indicating a lateral CoP displacement. The warm colours, red and orange, indicate high pressure; red represents the highest pressure, while cool colours, green and blue, indicate low pressure.

The study was undertaken at designated social running race events, which were selected based on their ability to produce enough participants who fulfilled the inclusion criteria. Participants were requested to walk barefoot across the Freemed™ 6050 force plate platform, which is a high-resolution system designed to capture dynamic plantar pressure distribution and CoP trajectory [18]. Each participant walked 22 times, with 11 steps per foot across the platform, ensuring adequate familiarisation with the setup to mitigate any variability due to discomfort or unfamiliarity. The rationale for 22 passes was to obtain a sufficient number of trials for both feet to enhance reliability, and the final CoP trajectory values were calculated as the mean of the valid trials for each foot. The CoP trajectory was recorded for each foot separately during the stance phase of gait (Figure 1).

### 2.4. Data Analysis

Data analysis primarily involved using cross tabulations to examine the relationship between participant characteristics (e.g., age, weight, foot type, load distribution) and the observed CoP trajectories. This allowed for comparing variables such as the degree of pes planus (flexible vs. rigid) and its impact on the CoP trajectory.

Chi-square tests were applied to assess the statistical significance of the relationships between categorical variables. In cases where expected frequencies were below 5, Fisher’s exact test was used to ensure the accuracy of the analysis. These tests were used to identify whether there were significant differences in load distribution and CoP patterns between the left and right feet and between individuals with different classifications of pes planus.

The level of statistical significance was set at *p* < 0.05 for all tests, and where multiple comparisons were made, Bonferroni corrections were applied to adjust for the increased risk of Type I error. To enhance interpretability, 95% confidence intervals (CIs) were reported alongside *p*-values for key findings where applicable, to provide an estimate of the precision of the observed effects. To ensure the reliability of the measurements, both interrater and intra-rater reliability were assessed during data analysis. Interrater reliability was evaluated by having two independent assessors review a subset of CoP trajectory data. The results were compared using Cohen’s kappa statistic, and a high level of agreement (kappa > 0.633) was obtained, indicating substantial agreement between raters. Intra-rater reliability was also evaluated. A subset of participants’ data was re-analysed by the primary rater after a two-week interval. The intra-rater reliability was similarly calculated using Cohen’s kappa statistic, with values above 0.85 indicating excellent reproducibility of the measurements. It is important to note that findings described as “notable” CoP displacements were interpreted descriptively unless supported by statistical significance, to avoid overstatement. Potential biomechanical explanations for the observed asymmetries in CoP trajectory between the right and left feet include unmeasured variables such as leg dominance and asymmetries in training load, which were not controlled for in this study. All analyses were conducted using IBM SPSS Statistics version 28, and results were presented in the form of cross-tabulation tables, with associated Chi-square or Fisher’s exact *p*-values to highlight any significant findings. From a clinical perspective, a lateral shift in CoP, particularly when recurrent, may alter lower limb biomechanics in a way that increases the risk of lateral ankle sprains or impairs shock absorption efficiency during ground contact. These alterations may inform not only orthotic design but also injury prevention strategies in runners with pes planus.

## 3. Results

### 3.1. Participant Characteristics

**Figure 2:**
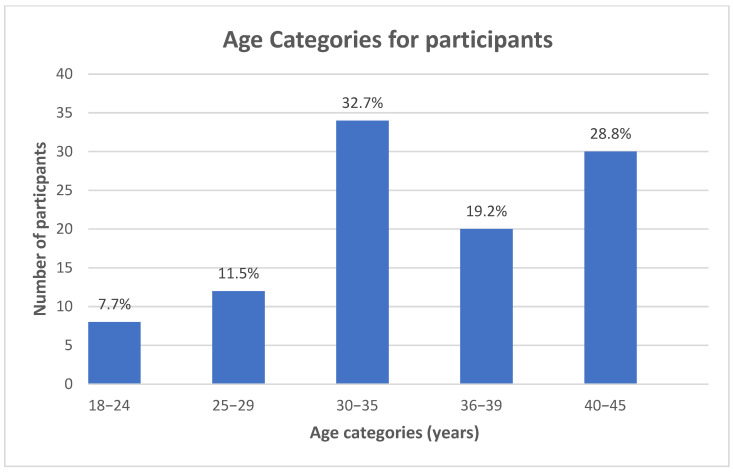
Age of participants. The three largest age groups were 30–35 years, 40–45 years, and 36–39 years. A total of 104 participants were included in the sample.

**Figure 3:**
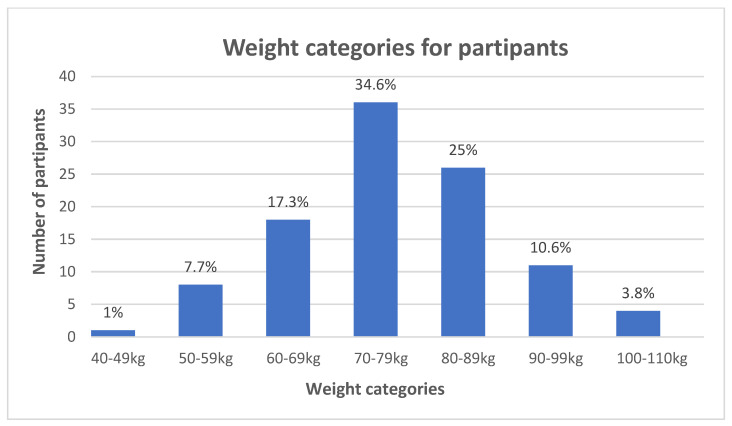
Weight of participants. The three largest groups were 70–79 kg, 89–89 kg, and 60–69 kg. A total of 104 participants were included in the sample.

After application of the inclusion and exclusion criteria, 104 participants were included in the study (N = 104). The largest age group in the study comprised participants aged 30 to 35 years, followed by those in the 40 to 45 age range (Figure 2). Regarding body weight, most participants fell into the 70 to 79 kg category, with a significant proportion also in the 80 to 89 kg range (Figure 3). There was no statistically significant difference between the foot type of the right foot and the weight range of participants (X^2^ = 5.721; df = 6; *p* = 0.455). Similarly, there was no statistically significant difference between the foot type of the left foot and the weight range of participants (X^2^ = 5.733; df = 5; *p* = 0.456).

### 3.2. Foot Posture Index

**Figure 4:**
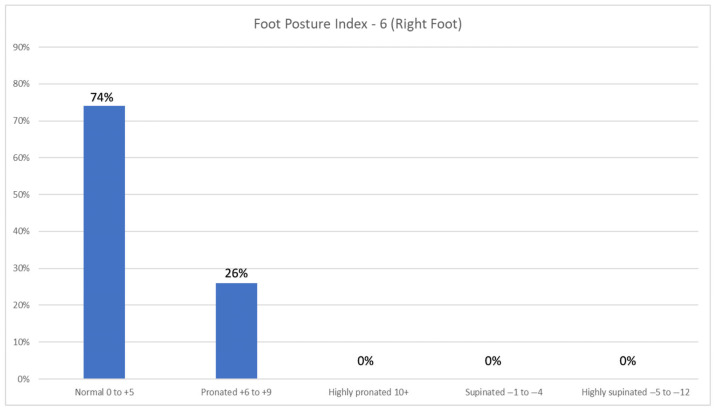
Foot posture index −6 of the right feet of participants. The majority had a normal foot posture index range of 0 to +5 for the right foot. A total of 104 participants were included in the sample.

**Figure 5:**
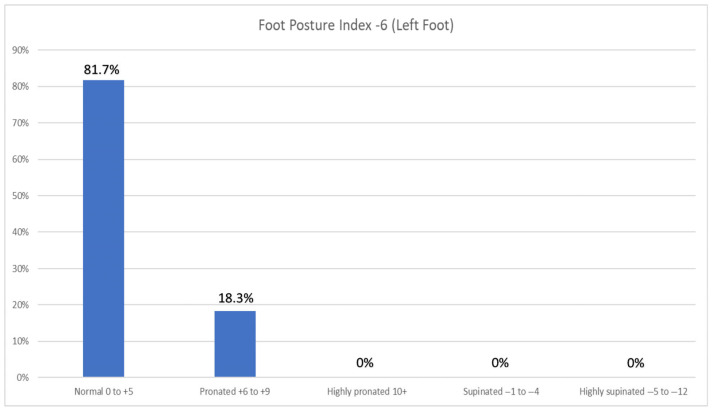
Foot posture index −6 of the left feet of participants. The majority had a normal foot posture index range of 0 to +5 for the left foot. A total of 104 participants were included in the sample.

The FPI-6 results of the right foot revealed that a significant majority of participants, 74.0% (n = 77), exhibited a normal foot posture, falling within the range of 0 to +5 on the FPI-6 scale. However, a notable proportion of 26.0% (n = 27) demonstrated a pronated foot posture within the range of +6 to +9. The left foot revealed that most participants (81.7%; n = 85) exhibit a normal foot posture ranging from 0 to +5 on the FPI-6 scale. In contrast, 18.3% (n = 19) of participants demonstrate a pronated foot posture falling within the range of +6 to +9 (Figure 4 and Figure 5). There was no statistically significant difference between the FPI-6 of the right foot and the weight range (X^2^ = 7.922; df = 6; *p* = 0.244).

### 3.3. Foot Type

**Figure 6:**
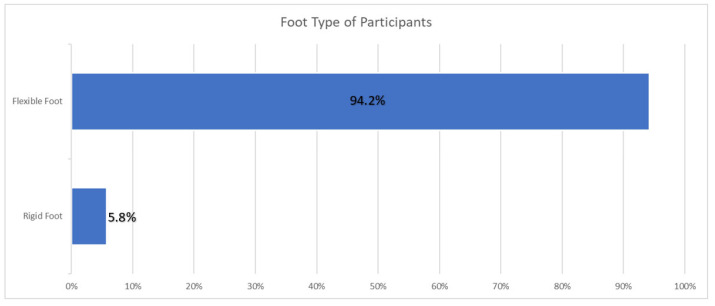
The foot type of participants. The majority of participants had flexible pes planus. A total of 104 participants were included in the sample.

Foot-type analysis showed that 94.2% (n = 98) of participants had flexible pes planus, while the remaining 5.8% (n = 6) presented with a rigid form of the condition (Figure 6). There was no statistically significant difference between the foot type of the right foot and the CoP of the right foot when using Fisher’s exact test (*p* = 0.463). Additionally, there is no statistically significant difference between the foot type of the right foot and the CoP of the left foot using Fisher’s exact test (*p* = 0.191).

### 3.4. Centre of Pressure Trajectory

Contrary to traditional expectations, the forefoot CoP trajectory demonstrated a predominant lateral displacement pattern rather than the anticipated medial displacement associated with overpronation [11]. The findings revealed a lateral displacement of the CoP in the forefoot in 90.4% (n = 94) of the participants for the right foot and 57.7% (n = 60) for the left foot. No statistically significant differences were found between the foot type for the left foot and the CoP of the (1) right foot and the (2) left foot when using Fisher’s exact test (*p* = 0.463 and *p* = 0.397, respectively).

### 3.5. Load Distribution

Analysis of load distribution revealed that participants exhibited asymmetry, with higher lateral loading on the right foot in most cases. The results indicated that the medial heel of the right foot bore the most load (34.6%), followed by the medial arch (26.9%) and the second and third metatarsal heads (19.2%). This suggests that the right foot primarily supports a higher load in its posterior and medial portions. In contrast, the left foot exhibited a more balanced load distribution, with the lateral heel (32.7%) and medial heel (20.2%) absorbing most of the stress, and a significant contribution from the lateral arch (18.3%). No statistically significant difference between the foot type of the right foot and the load percentage of the right foot (X^2^ = 7.326; df = 5; *p* = 0.197). Similarly, there is no statistically significant difference between the foot type of the right foot and the load percentage of the left foot (X^2^ = 5.724; df = 7; *p* = 0.572).

Tables presenting the data for foot type and centre of pressure. A cross-tabulation was conducted to evaluate the relationship between foot type and CoP trajectory for both right and left feet. Table 1 and Table 2 present data for the right foot and left foot, showing that the majority of participants had a lateral CoP trajectory.

**Table 1:** Cross-tabulation between foot type (right foot) and centre of pressure (right foot).

Foot Type (Right Foot)	Centre of Pressure (Right Foot): Medial	Lateral	Total
Rigid Foot Count (n)	1	5	6
% within CoP	10.0%	5.3%	5.8%
Flexible Foot Count (n)	9	89	98
% within CoP	90.0%	94.7%	94.2%
Total Count (n)	10	94	104
% within CoP	100.0%	100.0%	100.0%

**Fisher’s Exact Test**

- Exact Sig. (2-sided): 0.463
- Exact Sig. (1-sided): 0.463

**Table 2:** Cross-tabulation between foot type (left foot) and centre of pressure (left foot).

Foot Type (Left Foot)	Centre of Pressure (Left Foot): Medial	Lateral	Total
Rigid Foot Count (n)	1	5	6
% within CoP	2.3%	8.3%	5.8%
Flexible Foot Count (n)	43	55	98
% within CoP	97.7%	91.7%	94.2%
Total Count (n)	44	60	104
% within CoP	100.0%	100.0%	100.0%

**Fisher’s Exact Test**

- Exact Sig. (2-sided): 0.397
- Exact Sig. (1-sided): 0.191

## 4. Discussion

### 4.1. Interpretation of Findings

The study did not find a significant link between weight and foot posture, suggesting that weight may not substantially influence these parameters. This finding contrasts with some studies that have suggested a correlation between higher body weight and the development of pes planus [24]. The lack of a significant relationship in this study could be due to the specific characteristics of the sample population or other confounding factors such as activity level, muscle strength, and genetic predispositions. This highlights the need for further research to clarify the role of weight in the aetiology of pes planus. The hypothesis that Black African runners with pes planus would exhibit a lateral forefoot CoP trajectory was confirmed by the findings, supporting the existence of a distinct compensatory gait pattern in this population. However, the hypothesis regarding a relationship between body weight and foot posture was not supported, as no significant association was observed.

The results of this study indicated a higher prevalence of flexible pes planus than rigid pes planus. This high prevalence of flexible pes planus aligns with previous research findings. For instance, Raj et al. (2020) examined 138 athletes and found that 20% (n = 28) had pes planus, with all 28 athletes exhibiting the flexible type. Similarly, a study on 323 students revealed that 32.8% (n = 106) had pes planus, of which 89.62% had flexible pes planus [3]. These findings consistently demonstrate that flexible pes planus is a more common morphology than rigid pes planus [3].

The primary aim of the study was to investigate the CoP in the forefoot during barefoot gait in Black African runners with pes planus. The findings revealed a lateral displacement of the CoP in the forefoot of participants. These results align with those of De Cock et al. (2008) [15], who observed a similar lateral shift in CoP among barefoot runners with pes planus. Furthermore, Han et al. (2011) [25] investigated the CoP pathway in individuals with normal feet compared to those with pes planus, noting a medial shift in the forefoot CoP for normal arches and a lateral shift for pes planus. A study by Stolwijk et al. (2013) [19] compared CoP between Malawian and Dutch adults and found that the CoP was more laterally located in the forefoot leading to toe-off in Malawian participants. This observation is consistent with the findings of the present study, reinforcing the notion that the CoP in the forefoot of individuals with pes planus tends to be laterally displaced [25]. This lateral CoP course suggests that Black African runners with pes planus may adopt a unique compensatory gait strategy to maintain balance and propulsion. The observed lateral CoP pattern could be attributed to specific anatomical or neuromuscular characteristics unique to this population, which may influence their gait biomechanics differently than what is typically observed in Caucasian cohorts. However, caution must be exercised when generalising these findings to all Black African populations, as this study’s sample was regionally specific. The study’s findings challenge the conventional understanding of pes planus, leading to excessive medial CoP displacement and overpronation. The lateral CoP trajectory identified suggests a need to reconsider how we interpret and treat pes planus, particularly in Black African populations.

The study revealed distinct load-distribution patterns between the left and right feet of participants, indicating anatomical and functional differences. The right foot showed higher load concentrations in the medial heel and arch, whereas the left foot exhibited a more balanced distribution. These findings align with Han et al. (2011) [25], who noted plantar pressure distribution variations among pes planus individuals. Understanding these load distribution patterns is important for designing effective orthotic devices and rehabilitation programs aimed at redistributing pressure and reducing the risk of injury [21]. This asymmetrical loading could predispose runners to specific injuries, such as lateral ankle sprains or stress fractures, especially in the presence of repetitive gait cycles without adequate biomechanical control or compensation [22].

### 4.2. Comparison with Existing Literature

Previous studies have primarily focused on Caucasian populations, often concluding that pes planus is synonymous with overpronation and medial CoP displacement [10]. However, studies involving other ethnic groups have indicated variations in foot structure and gait biomechanics, suggesting that pes planus does not universally result in overpronation during gait [15]. The study suggests that the biomechanical effects of pes planus can vary significantly based on ethnicity and other demographic factors [23]. The lateral CoP trajectory observed in this study may indicate a compensatory mechanism that reduces the risk of medial stress but could increase the likelihood of lateral compartment injuries. This study’s findings contribute to a growing body of literature that calls for a more individualized approach to assessing and managing pes planus, considering factors such as ethnicity, foot morphology, and gait characteristics.

### 4.3. Clinical Implications

This study enhances our understanding of the unique biomechanical effects of pes planus on the males of the Black African population, an area previously lacking in research. It found that most participants in this group exhibited supination during barefoot gait, which contradicts the well-known adage that pes planus equates to overpronation. This finding underscores the relevance of studying this population, as generic advice focused on overpronation would not have been beneficial. Conventional orthotics designed to correct overpronation by supporting the medial arch may not be suitable for all individuals with pes planus, particularly in populations exhibiting a lateral CoP trajectory. This highlights the need for clinicians to reassess their methods for evaluating and managing pes planus, including their use of orthotics, footwear prescription, and exercises. This study’s insights into CoP displacement provide a foundation for future research, aiming to improve patient outcomes and advance clinical practice. In addition to influencing orthotic design, the lateral CoP pattern identified in this study may also increase susceptibility to lateral ankle sprains and impair shock absorption, underlining the importance of comprehensive injury prevention strategies.

### 4.4. Limitations and Future Research

This research involved only male participants to account for biomechanical and physiological variations between genders. However, this limits the generalizability of the results to encompass a border population. All participants were of the Black African population affinity, due to specific population prevalence and research paucity. However, this restricts the applicability of the results to other population affinity groups. Although the sample size of 104 participants meets the requirements for robust statistical analysis. A greater sample size could produce more generalizable findings and enable the detection of a smaller effect size. Additional limitations include the absence of a control group comprising Black African runners without pes planus, which would have enhanced comparative analysis. Moreover, the study focused exclusively on walking rather than running gait, which limits conclusions regarding dynamic functional performance under typical training or competitive conditions. The repeated walking trials (22 per participant) may also have introduced learning or fatigue effects that could influence CoP trajectory or load distribution, despite efforts to standardise participant familiarisation. Future research should incorporate more advanced biomechanical tools such as 3D gait analysis, electromyography, or kinetic modelling to provide deeper insights into muscular coordination and joint forces associated with pes planus gait.

Future research should include larger cohorts and explore the role of other variables, such as muscle strength, flexibility, and footwear, in influencing CoP trajectory and load distribution. Additionally, longitudinal studies examining the impact of different interventions on CoP trajectory and injury rates in female Black African runners with pes planus are needed to further validate these findings.

## 5. Conclusions

This study provides new insights into the biomechanics of pes planus in Black African recreational runners, highlighting a lateral CoP trajectory that challenges traditional views of overpronation. These findings suggest more individualized assessments and interventions for pes planus, particularly in diverse populations. Understanding these unique gait characteristics will help clinicians develop targeted strategies to manage pes planus effectively, reduce injury risk, and enhance the performance of recreational runners. However, findings should be interpreted within the context of this study’s specific sample and design, and not extrapolated to the entire Black African population without further investigation.

## Data Availability

Data supporting reported results can be requested directly from Yaasirah Mohomed Choonara at yaasirahc@uj.ac.za.

## Abbreviations

The following abbreviations are used in this manuscript:

CoP	Centre of Pressure
3D	Three-dimensional
FPI-6	Foot Posture Index-6
